# The Role of Explicit and Implicit Self-Esteem in Peer Modeling of Palatable Food Intake: A Study on Social Media Interaction among Youngsters

**DOI:** 10.1371/journal.pone.0072481

**Published:** 2013-08-28

**Authors:** Kirsten E. Bevelander, Doeschka J. Anschütz, Daan H. M. Creemers, Marloes Kleinjan, Rutger C. M. E. Engels

**Affiliations:** Behavioural Science Institute, Radboud University Nijmegen, Nijmegen, The Netherlands; University of Missouri-Kansas City, United States of America

## Abstract

**Objective:**

This experimental study investigated the impact of peers on palatable food intake of youngsters within a social media setting. To determine whether this effect was moderated by self-esteem, the present study examined the roles of global explicit self-esteem (ESE), body esteem (BE) and implicit self-esteem (ISE).

**Methods:**

Participants (N = 118; 38.1% boys; *M* age 11.14±.79) were asked to play a computer game while they believed to interact online with a same-sex normal-weight remote confederate (i.e., instructed peer) who ate either nothing, a small or large amount of candy.

**Results:**

Participants modeled the candy intake of peers via a social media interaction, but this was qualified by their self-esteem. Participants with higher ISE adjusted their candy intake to that of a peer more closely than those with lower ISE when the confederate ate nothing compared to when eating a modest (*β* = .26, *p* = .05) or considerable amount of candy (kcal) (*β* = .32, *p* = .001). In contrast, participants with lower BE modeled peer intake more than those with higher BE when eating nothing compared to a considerable amount of candy (kcal) (*β = *.21, *p* = .02); ESE did not moderate social modeling behavior. In addition, participants with higher discrepant or “damaged” self-esteem (i.e., high ISE and low ESE) modeled peer intake more when the peer ate nothing or a modest amount compared to a substantial amount of candy (kcal) (*β* = −.24, *p* = .004; *β* = −.26, *p*<.0001, respectively).

**Conclusion:**

Youngsters conform to the amount of palatable food eaten by peers through social media interaction. Those with lower body esteem or damaged self-esteem may be more at risk to peer influences on food intake.

## Introduction

Computer use has been associated with increased sedentary behavior as well as (soft) drink and snack consumption among youngsters, which can contribute to being overweight [1], [2]. The majority of Dutch youth are on the Internet (96%) and converse by social media (e.g. MSN, Skype, Face book) for approximately 1.5 hours a day [3], [4]. As friends and peers become more important with age, the amount of time spent on social media increases significantly during high school [3]. Numerous experimental studies have shown by means of “confederates,” who were secretly instructed to choose or eat certain types or amounts of food, that individuals adapt the food intake of peers [5], [6], [7]. This so-called social modeling effect was found regardless of whether the confederates were physically present (i.e., “remote” or “video” confederates) and illustrates the strong influence of others on food consumption [8], [9], [10], [11]. For example, boys and girls were found to follow a remote confederate’s unfamiliar food choices during a computer game while they were shown food choices between familiar and unfamiliar foods on screen [12]. In addition, a study among girls showed that they consumed more after seeing a remote (video) confederate eat a large rather than a small amount of palatable food [13]. It is unknown whether a remote confederate also influences consumption when youngsters engage in an online social interaction.

Social modeling behavior is based on a normative framework; that is, people use others’ food intake as a norm or guideline for how much is appropriate to eat [14], [15]. From infancy on, people model their behaviors to learn and to affiliate with others as well as to be liked and socially embedded due to our need to belong [16], [17]. However, individual characteristics [18] and social context affect to what extent people adjust their food intake [15]. For example, a study of young adults showed that females only followed the food intake of a real confederate when she was acting less sociable [19]. The authors argued that the participants felt a stronger need to affiliate when the confederate was acting “socially cold” than when she was acting “socially warm,” because the affiliation goal had been already achieved for the latter.

Social belonging is determined in part by self-esteem [20], [21] and self-esteem plays an important role in social interactions [22]. According to the sociometer theory, self-esteem can be seen as a monitor of social acceptance and exclusion [22]. People with high self-esteem are more likely to believe that others like them than people with low self-esteem [23], [24]; they worry less about how they are perceived by others and perceive a lower probability of rejection [20]. Subsequently, people with high self-esteem feel less need to affiliate with others and to affirm social bonds (e.g., by social modeling) compared to people with low self-esteem [16], [20], [25]. Because individuals model behavior to affiliate or fit in [16], [17], self-esteem may also play a role in social modeling of food intake. To our knowledge, there is only one study that examined the role of self-esteem on the matching degree of food intake in female students. Robinson et al. [26] found strong matching in dyads where one co-eater had low self-esteem but no matching effect in dyads where both co-eaters had high self-esteem. However, it was not possible to infer whether the participant with low self-esteem matched the food intake of the co-eater with high self-esteem, or vice versa. The present study aimed to address the question of causality.

Furthermore, it is important to note that the construct of self-esteem can be assessed in various ways. Most literature deals with global explicit self-esteem (ESE), which assesses people’s positive or negative attitude toward the self as a totality. While ESE provides insight into general psychological well-being, eating behavior might be better explained by domain-specific self-esteem (e.g. academic performance, athletic competence or (body) appearance) [27], [28], [29], [30]. In line with this notion, having low body esteem was previously found to predict low global ESE, but not vice versa [27], [31]. As research showed that young people’s body esteem is related to their eating behaviors [32], the current study also included body esteem (BE) as a explicit domain-specific measure of self-esteem.

The construct of self-esteem can be further distinguished by taking into account implicit self-esteem (ISE). ISE is based on intuitive automatic self-evaluations, whereas ESE is based upon a conscious effortful retrieval of information to evaluate the self. It has been proposed that ISE develops early in life, which would produce a pre-conscious affective response to self-relevant stimuli by drawing on associative links in memory [33]. In contrast, ESE is likely to be constructed as a function of specific contexts and goals by drawing on cognitive capacity. A new line of research investigates the discrepancy between ESE and ISE. For example, a high ISE but low ESE (i.e. “damaged” self-esteem) is related to people’s (disturbed) eating behavior [34]. It has been proposed that ISE might reflect a presentation of the ideal self, whereas ESE represents the real self, and that the discrepancy could lead to a disturbed feeling [35]. Therefore, a discrepancy between ESE and ISE might be seen as an indicator of psychological distress that can create uncertainty and lead to difficulties in maintaining a consistent self-view, which subsequently results in lower levels of mental and physical health [35], [36]. To our knowledge, the influence of ISE or a possible discrepancy between ESE and ISE on social modeling behavior of food intake has not yet been examined.

The aim of the present study is to investigate whether the palatable food intake of a peer (i.e., remote confederate) had an effect on the food intake of youngsters via social media interaction and whether this influence depended upon ESE, BE, ISE or a discrepancy between ESE and ISE. It was hypothesized that youngsters adjust their food intake to that of a peer but that those with lower ESE would follow the food intake of a peer more closely than those with higher ESE. Similar effects were hypothesized for BE, but it was expected that BE would have a stronger impact on modeling of food intake than ESE. As this is the first study to include the role of ISE on social modeling behavior, it explored whether ISE or a possible discrepancy between ESE and ISE had an effect on peer modeling of eating.

## Methods

### Participants

**Figure 1:**
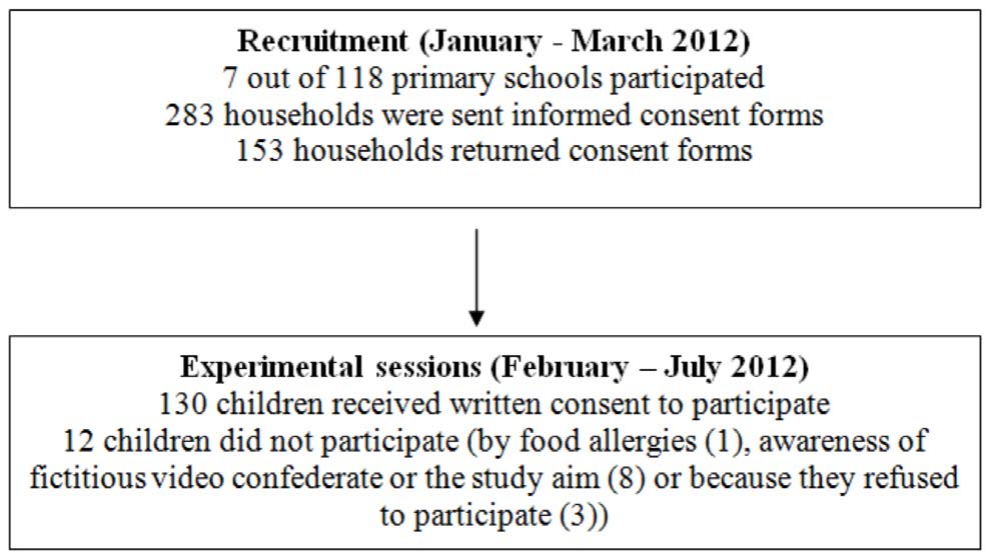
Flow diagram of the recruitment procedure.

Figure 1 depicts a flow diagram of the recruitment procedure for the study. School teachers of grades 5 and 6 distributed detailed consent forms to parents of the students. For all schools that participated in this study, more than 70% of the students had a West-European or Dutch background. The study sample consisted of 118 participants (38.1% boys) with a mean age (SD) of 10.53 years (.54) in grade 5 (n = 49) and 11.58 years (.63) (n = 69) in grade 6. Most participants (85.6%) were normal weight; 8.5% were overweight and 5.9% were underweight. The present study was conducted according to the guidelines of the Declaration of Helsinki, and procedures were approved by the Ethics Committee of the Faculty of Social Sciences, Radboud University Nijmegen. Active written informed consent was obtained from the student’s caregivers.

Power calculations were conducted using the program G*Power 3.1.2 [37]. To detect a medium to large effect using linear regression (f^2^ = 0.20) with 7 predictors (it was estimated that besides the inclusion of the main variables - self-esteem and the intake conditions - the control variables food liking, hunger and BMI had to be included), 80 participants are needed (power 0.80, *p*<.05). Taking into account the dependence of measurement within school classes, we followed the procedure proposed by Twisk [38] with an estimated Intra Class Correlation (ICC) equaling.04. The number of students was estimated on 15 students per class who would receive written consent by their parents and this resulted in a multiplier of 1.5. Therefore, 120 pupils in total had to be approached. However, more than 120 students were recruited because it was expected that some parents would not give informed consent or participants had to be excluded due to the study design.

### Setting and Procedure

**Figure 2:**
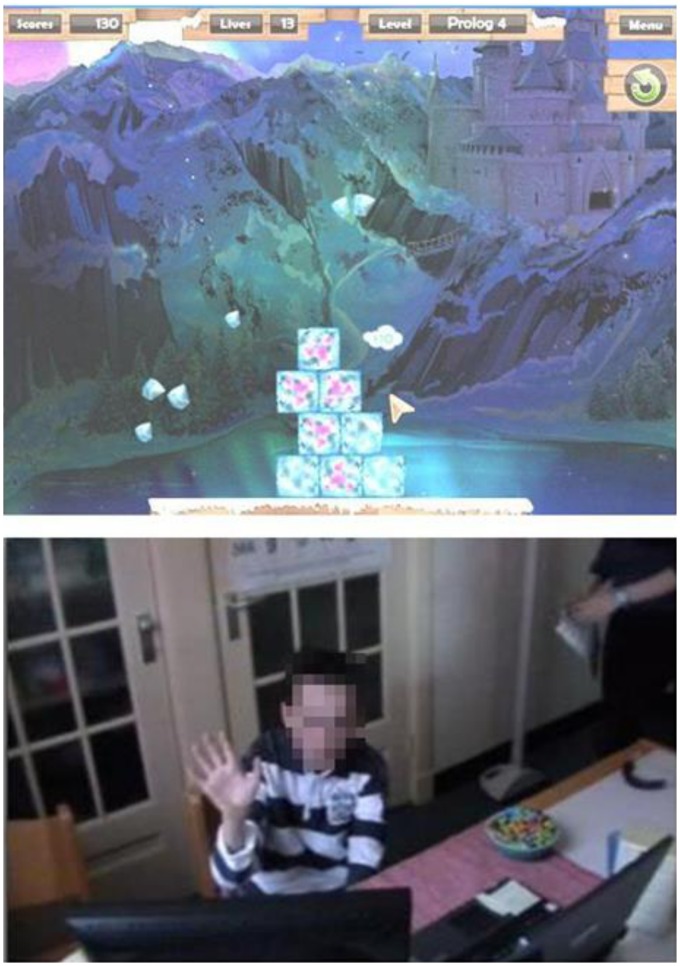
Computer game “Shooting Blocks” (above) and a participant waving good-bye to the remote confederate at the end of the online interaction (below).

Data collection took place from February through June 2012 between 8∶30 AM and 3∶30 PM. The social media interaction lasted 10 minutes and was videotaped. The video camera was placed on a tripod in front of the participants, which they thought was used as a web cam. Participants were seated at a table with a laptop to play the computer game, a glass of water and a bowl of candy (i.e., chocolate-coated rice crisps). A large computer screen and sound speakers (connected to a second laptop) were placed next to the participant’s laptop, through which they were able to see and hear the remote confederate. Figure 2 presents a still of the computer game and the setting of the study. The computer game (“shooting blocks”) consisted of different levels with constructions (e.g., a tower or pyramid) composed of ice cubicles, some of which were pink. The participants could earn points by breaking the pink ice cubes with the computer mouse. They had to start the level over again if the construction collapsed and too many non-pink ice cubs were lost.

#### Experimental intake conditions and remote confederates

The remote confederates were young teenagers who were trained at a drama academy. There were three male and three female normal-weight confederates who were videotaped for each experimental intake condition. Acting according to the same script, they made remarks about the computer game, asked questions, and gave helpful instructions. Similar to previous research, they were instructed to eat nothing (no-intake condition), four pieces of candy (low-intake condition), or 15 pieces of candy (high-intake condition) at set time points which were signaled by use of a buzzer device [18]. The remote confederates ate the same type of candy as the participants. The participants were randomly assigned to one of the experimental (no-, low- and high-) intake conditions.

#### Cover story and modeling experiment

The participants were delivered a cover story to conceal the actual aim of the study. Before starting the experiments in school, each class was told that the experimenters were interested in computer gaming with another peer and that an average score would be calculated by their game score and the score of another peer who was playing at the same time but at another school. Prior to the social modeling experiment, the participants were told that they had to wait for the remote confederate to come and play the computer game. They were asked to complete some computer tasks (i.e. the self-esteem measures) while they were waiting. After they finished the self-esteem measures, one experimenter made the video connection (i.e., started the video clip with the remote confederate), while the other experimenter instructed the participant about the computer game. At the same time, the participant could see and hear that the remote confederate received the same instruction by another experimenter (i.e., an actor). The experimenter made sure to wave with the participant to their remote counterparts at the exact moment that the latter waved on the video. To conceal that the participants could not really interact with the remote confederate, the participants were told that there were problems with the sound connection at the other school. Nevertheless, the participants were encouraged to try to interact whether or not the sound was working. The experimenter left the room at the same moment as the experimenter did on the video. After exactly 10 minutes, the experimenter came back again (similar to the video), waved to the remote counterparts and switched off the electronic devices. The participants’ height and weight were measured, and a short questionnaire was administered.

### Measures

#### Food intake participant

The experimenter weighed the bowls of candy before and after each session using a digital scale (Kern 440, Kern & Sohn, Balingen, Germany). The consumed grams were converted into kilocalories (100 gr/471 kcal) and used as the dependent variable in the analyses.

#### Explicit self-esteem

Explicit self-esteem (ESE) was assessed by the Rosenberg self-esteem scale which is a widely used 10-item self-report measure of self-esteem. Participants rated the items (e.g., “On the whole, I am satisfied with myself”) on a scale from 1 (strongly agree) to 4 (strongly disagree). Cronbach’s alpha was α = .80.

#### Body esteem

The participant’s body esteem (BE) was measured by the Children Figure Rating Scale, which consists of nine children’s appearance drawings ranging from very thin (1) to obese (9) [39]. The participants were asked to choose the drawing which they perceived their current figure to be and which they perceived as their ideal figure to be. The discrepancy between their perceived current figure and their ideal figure represented their BE [40]. The higher the score, the greater their body dissatisfaction and the lower their BE [32]. As it has been suggested that people who want to gain weight might have a different BE than people who want to lose weight [32], [41], BE was additionally tested by recoding the participant’s score of who wanted to gain weight as missing score.

#### Implicit self-esteem

Implicit self-esteem (ISE) was assessed with the Implicit Association Task (IAT) [42]. The IAT measures the positive and negative associations that an individual has with the self and with others. It is a computer-based response time task in which participants categorize stimuli by rapidly pressing a left-side or right-side key on the laptop keyboard without making errors. The reaction time measure assesses the relative difference of association between two target categories (i.e., me vs. not-me) with two attribution categories (i.e., positive vs. negative words or attitudes). The measure is computed by the speed at which participants press the keys in which association strengths influence performance. Participants respond faster to highly associated categories (e.g., me+positive attributions) than to less associated categories (e.g., she+positive attributions or me+negative attributions). Thus, the scores reflect the ease with which participants associate positive versus negative words with the self. The overall IAT score is computed by taking the difference between the average response times for the two test blocks (blocks 4 and 7, which were counterbalanced across participants to control for order effects). The degree to which “me-positive” and “not-me-negative” are stronger associations than “me-negative” and “not-me-positive” indicates more implicit self-esteem (see Table 1 for an overview of the IAT task). The improved scoring algorithm was used (*D*-measure) to compute individual scores as the difference in mean latencies between the two test blocks, divided by the inclusive standard deviation of trials within the respective blocks (for further specific details on the *D*-measure such as practice trials and exclusion criteria, see Greenwald et al. [43]). The IAT was programmed in Inquisit 3.0 (Millisecond software).

**Table 1:** Procedure of the IAT response task.^1^

Block		No. of trials	Left response key ‘E’	Right response key ‘I’
1	Practice	20	Me	Not-me
2	Practice	20	Positive attributions	Negative attributions
3	Practice	40	Me+positive attributions	Not-me+negative attributions
4	Test	40	Me+positive attributions	Not-me+negative attributions
5	Practice	20	Not-me	Me
6	Practice	20	Not-me+positive attributions	Me+negative attributions
7	Test	40	Not-me+positive attributions	Me+negative attributions

1 The 2 target categories were: I, Me, My, Myself, Self, Mine versus His, Her, They, Them, Their, Others. Positive versus negative attribution categories were: Fun, Nice, Positive, Good, Worthy, Clever versus Pathetic, Stupid, Negative, Bad, Worthless, Unintelligent (In Dutch these words were translated as: Leuk, Aardig, Positief, Goed, Waardevol, Slim versus Onaardig, Stom, Negatief, Slecht, Waardeloos, Dom).

#### Body weight

Body weight is controlled for in the analyses as it is associated with BE and social modeling behavior [18]. The experimenter measured height and body weight individually according to standard procedures (without shoes but fully clothed). Height was measured to the nearest 0.1 cm using a stadiometer (Seca 217 Slider, Seca GmbH & Co., Hamburg, Germany) and weight was measured to the nearest 0.1 kg using a digital scale (Seca Bella 840, Seca GmbH & Co.). The body mass index (BMI) was calculated using the formula: weight [kg]/height2 [m]. BMI (z-score) cutoff points which are representative of current z-BMI standards for Dutch youngsters were used [44].

### Measurements Questionnaire

#### Hunger

To conceal the real aim of the study, participants’ subjective hunger state was measured after the experiment. The participants indicated their hunger on a Visual Analogue Scale (VAS) (0 cm, *not hungry at all*; 15 cm, *very hungry*) [18].

#### Time of day

Participants’ food intake might be related to time of day. Afternoons are more commonly snack times than mornings [45]. Therefore, the actual time of day on which the participant played the computer game during the online social interaction was taken into account.

#### Liking of the candy

Liking of the candy was previously found to affect the participants’ food intake [18]. The participants were asked to indicate how much they liked the candy on a VAS (0 cm, *not at all*; 15 cm, *very much*) with a sad looking and smiley face at the start and end of the scale, respectively.

#### Liking of the task

To measure the extent to which the participants liked the computer game, a VAS was used (0 cm, *do not like at all*; 15 cm, *like it a lot*) with a sad looking and smiley face at the start and end of the scale, respectively.

#### Liking of the remote confederate

Liking of the remote confederate might influence food intake. To measure the extent to which the participants liked the remote confederate, a VAS was used (0 cm, *do not like at all*; 15 cm, *like him/her a lot*) with a sad looking and smiley face at the start and end of the scale, respectively.

#### Estimation of the remote confederate’s candy intake

To test whether the participants were conscious of the remote confederate’s candy intake, they were asked if they could estimate his/her candy intake (expressed in the number of candies).

### Analytical Strategy

Data were analyzed using SPSS for Windows (version 20.0, 2012, SPSS Inc., Chicago, IL, US). Alpha was set at *p<*.05. First, randomization checks were performed by using one-factor analysis of variance to test for differences among the three experimental intake conditions. Second, Spearman’s rank and Pearson’s correlations were performed for the model variables of age, sex, hunger, liking of the candy, time of day the experiment took place, liking of the task, liking of the remote confederate and candy intake (kcal) to determine which variable had to be controlled for in the main analyses.

The intraclass correlation coefficient (ICC) for the outcome variable candy intake (kcal) was.05 meaning that 5% could be explained by nestedness within schools. According to Muthén [46], the size of the effect should preferably be under 5%. To control for the possible impact of clustering within schools, analyses were conducted in MPLUS with a sampling design adjusted model with schools as clusters, using the Type is Complex option in Mplus 6.0 [47]. Of the 118 participants, 3 participants did not complete the ESE task and 5 participants did not complete the ISE task. For BE, 9 participants reported an ideal body shape that was larger than their current body shape. In a second analysis for BE, they were coded as ‘missing.’ Therefore, the analyses for ESE, ISE and BE were performed for N = 115, N = 113, N = 118 and N = 109 participants, respectively. Maximum percentage missing values was 7.6%. Missing values were handled in Mplus using full information maximum likelihood (FIML) estimation.

First, to examine whether social modeling occurred during social media interaction, the main effects of self-esteem and the experimental intake condition on candy intake (kcal) were tested in 2 different models by means of dummy coding the experimental intake conditions. Second, the interaction effects between the different self-esteem scores and experimental intake condition were tested. Model 1 tested no-intake as a reference group (dummy coded as 0) against the low-intake and high-intake condition (dummy coded as 1), and model 2 tested the low-intake as a reference group against the no-intake and high-intake condition. The interaction terms were calculated between the dummy variables (i.e., the experimental intake conditions) and the different types of self-esteem and entered into the models while controlling for hunger and liking of the candy. To interpret possible interaction effects plots were constructed using the unstandardized regression coefficients. Similar models were used to examine discrepancies between the implicit and explicit measures.

## Results

### Randomization and Manipulation Checks

Randomization checks were performed to test for differences between the experimental intake conditions in age, sex, hunger, liking of candy, liking of the task, liking of the remote confederate, ESE, ISE, BE. Table 2 summarizes the means and standard deviations (SDs) for all variables in each experimental intake condition. There were no significant differences (*P*>.10) between the experimental intake conditions, which indicated that randomization was successful.

**Table 2:** Randomization checks of the variables measured by experimental intake condition.^1^

Variables	No – intakeconfederate (*n* = 41)		Low – intakeconfederate (*n* = 36)		High – intakeconfederate (*n* = 41)		*P* value^2^
Age (y)	11.17 (.83)	10–13	11.08 (.81)	10–13	11.17 (.74)	10–12	.86
Boys/girls (*n*/*n*)	18/23		11/25		16/25		.49
BMI (*z*-score)	.32 (.92)	−1.78–3.62	.38 (1.33)	−4.13–2.98	.05 (.74)	−1.44–1.40	.30
Hunger	36.10 (29.16)	1–113	39.44 (34.76)	1–127	33.46 (27.47)	1–138	.69
Liking of candy	109.73 (35.64)	2–151	115.46 (33.06)	13–150	114.78 (36.98)	15–150	.73
Liking of task	114.80 (27.62)	38–150	122.88 (22.36)	51–149	110.22 (29.84)	42–150	.13
Liking remote confederate	115.70 (20.87)	57–150	119.11 (21.60)	60–150	117.71 (14.92)	93–150	.74
Time of day	11∶58 (1∶58)	8∶35–14∶55	11∶57 (1∶56)	8∶55–14∶50	11∶59 (1∶57)	9∶05–14∶40	.99
Global explicit SE	3.11 (.43)	1.80–3.80	3.11 (.40)	2.20–3.80	2.96 (.44)	1.80–3.80	.20
Body esteem	.48 (1.03)	−2–4	.42 (.69)	−2–2	.29 (1.03)	−2–3	.64
Implicit SE	.44 (.41)	−.33–1.11	.59 (.33)	−.64–1.30	.49 (.30)	−.20–.89	.17

1 Values are presented in means (SD), min. – max.

The manipulation check showed that there were significant differences (N = 117; *F*_2,115_ = 42.18, *p*<.001) in the participant’s estimations (1 participant did not provide an estimation) of the number of candies the remote confederate ate between the experimental intake conditions (no-intake: *M* = 1.17 (±2.31); low-intake: 6.94 (±4.67); high-intake: 13.88 (±9.42). Post hoc analysis with Bonferroni correction showed that the participants’ estimations were significantly different (*p*<.001) for the experimental intake conditions.

### Main Analyses

Spearman’s rank and Pearson’s correlations showed that age (*r* = .02, *p* = .79), sex (*r_s_* = .07, *p* = .48), time of day they played the computer game (*r* = .04, *p* = .67), liking of the task (*r* = .12, *p* = .19) and liking of the remote confederate (*r* = .10, *p* = .27) did not correlate significantly with candy intake (kcal). Hunger (*r* = .24, *p = *.009) and liking of the candy (*r* = .27, *p* = .003) were related to candy intake (kcal). Therefore, hunger and liking of the candy were entered into the models as covariates (in addition to BMI).

All Mplus models were saturated. In saturated models, all possible correlations between the independent variables and all possible direct paths from the predictors to the dependent variables are specified, so no fit measures are presented (Kline, 2011). The covariates hunger and liking of the candy had a significant effect on candy intake (kcal) in all three self-esteem measures in both models with model 1 testing no-intake versus low- and high-intake, and model 2 testing low- versus high-intake.

#### Explicit self esteem

The covariates hunger (*β = *.19, SE = .07, *p* = .006) and liking of the candy (*β = *.20, SE = .09, *p* = .036) had a significant effect on candy intake (kcal), and there were significant main effects of the experimental intake conditions on candy intake (kcal). Model 1 showed a significant difference between the no- and low-intake condition (*β = *.24, SE = .08, *p* = .002) and the no- and high-intake condition (*β = *.30, SE = .12, *p* = .013) on participant’s candy intake (kcal). Model 2 showed no significant differences between the low- and high-intake condition (*p* = .59). There were no effects of z-BMI (*p* = .41) or ESE (*p* = .76) on candy intake (kcal). There were also no significant interaction effects between ESE and experimental intake condition on candy intake (kcal) (*p*>.05).

#### Body esteem

The covariates hunger (*β = *.11, SE = .04, *p* = .001) and liking of the candy (*β = *.10, SE = .05, *p* = .028) had a significant effect on candy intake (kcal), and there were significant main effects of the experimental intake conditions on candy intake (kcal). Model 1 showed a significant difference between the no- and low-intake condition (*β = *9.46, SE = 2.89, *p* = .001) and the no- and high-intake condition (*β = *10.88, SE = 4.03, *p* = .007). Model 2 showed no significant differences between the low- and high-intake condition (*p* = .60). There were no effects of z-BMI (*p* = .71) or BE (*p* = .98) on candy intake (kcal).

**Figure 3:**
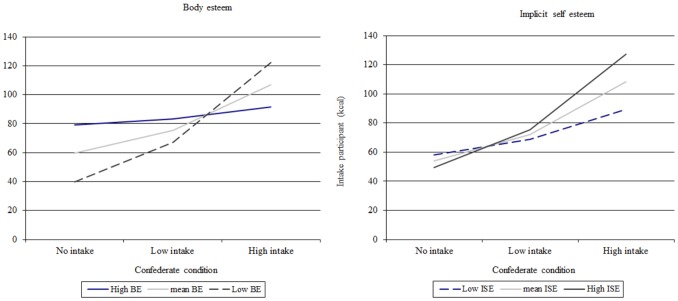
Interaction effects between experimental intake condition, ISE and BE on social modeling of candy intake (kcal). *Note:* The figure presents an interpretation of the interaction effect plotted with the unstandardized regression coefficients. In BE, there is a significant difference between the no- and high-intake condition for youngsters with lower BE. In ISE, there is a significant difference between the no- and high-, and low- and high-intake condition for those with higher ISE.

The main effect of the experimental intake condition on the participant’s candy intake (kcal) was qualified by an interaction effect between BE and experimental intake condition on participant’s candy intake (kcal). The standardized regression weights of the interaction models are presented in Table 3. There was only a significant difference between the no- versus high-intake condition (*β = *.21, *p* = .02). Figure 3 presents the interpretation of the interaction effects for BE. It shows that participants with lower BE followed the candy intake of the remote confederate more closely when they ate a substantial amount of candy compared to nothing. The models were also tested without the participants (*n* = 9) who wanted to gain weight. The models showed a significant difference between the no- versus high-intake condition (*β = *.26, *p* = .02) and between the low- versus high-intake condition (*β = *.43, *p* = .04) implying that participants with lower BE followed the candy intake of the remote confederate more closely when they ate nothing or a modest amount compared to a substantial amount of candy.

**Table 3:** Standardized parameter coefficients for the path models to test the interaction effects on candy intake (kcal).

Variables	ESE (N = 115)		ISE (N = 113)		BE (N = 118)	
Model 1	Coefficient	SE	Coefficient	SE	Coefficient	SE
Hunger status	.17*	.07	.21**	.08	.18*	.08
Liking candy	.19*	.10	.22*	.10	.21*	.09
BMI (z-score)	.04	.06	.06	.06	.07	.10
Self-esteem	.13	.18	−.10	.11	−.12	.15
Condition low intake^1^	.09	.64	.24	.14	.26*	.10
Condition high intake^1^	1.23	.80	.08	.18	.23	.14
Interaction no vs low*self-esteem	.17	.66	.07	.15	−.06	.11
Interaction no vs high*self-esteem	−.92	.86	.32**	.10	.21*	.09
Model 2						
Hunger status	.17*	.07	.21**	.08	.18*	.08
Liking candy	.19*	.10	.22*	.10	.21*	.09
BMI (z-score)	.04	.06	.06	.06	.07	.10
Self-esteem	.18	.14	−.03	.11	−.25	.25
Condition no intake^2^	−.09	.65	−.24	.14	−.27*	.11
Condition high intake^2^	1.14	.50*	−.16	.10	−.04	.14
Interaction low vs no*self-esteem	−.18	.68	−.06	.14	.09	.17
Interaction low vs high*self-esteem	−1.09†	.57	.26*	.13	.29	.21

Model 1 presents ‘no versus low and high intake condition’ and model 2 ‘low versus no and high intake condition’ for the self-esteem measures.

Note: † marginal significant p = .059, *p<.05, **p<.01.

1 Model 1: Reference is no intake versus low and high experimental intake condition.

2 Model 2: Reference is low intake versus no and high experimental intake condition.

#### Implicit self esteem

The covariates hunger (*β = *.19, SE = .07, *p* = .009) and liking of the candy (*β = *.20, SE = .09, *p* = .02) had a significant effect on candy intake (kcal), and there were significant main effects of the experimental intake condition on participant’s candy intake (kcal). Model 1 showed a significant difference between the no- and low-intake condition (*β = *.24, SE = .08, *p* = .003) and the no- and high-intake condition (*β = *.29, SE = .12, *p* = .012). Model 2 showed no significant differences between the low- and high-intake condition (*p* = .57). There were no main effects of z-BMI (*p* = .48) or ISE (*p* = .84) on candy intake (kcal).

Moreover, there was a significant interaction between ISE and the experimental intake condition on candy intake (kcal). The models showed a significant difference between the no- versus high-intake condition (*β* = .32, *p* = .001) and the low- versus high-intake condition (*β* = .26, *p* = .05). Figure 3 presents the interpretation of the interaction effects found between ISE and the experimental intake conditions. It shows that the participants with higher ISE followed the remote confederate’s candy intake more closely when they ate nothing or a modest amount compared to a substantial amount of candy.

### Additional Analyses on Implicit and Explicit Self-esteem Discrepancies

**Figure 4:**
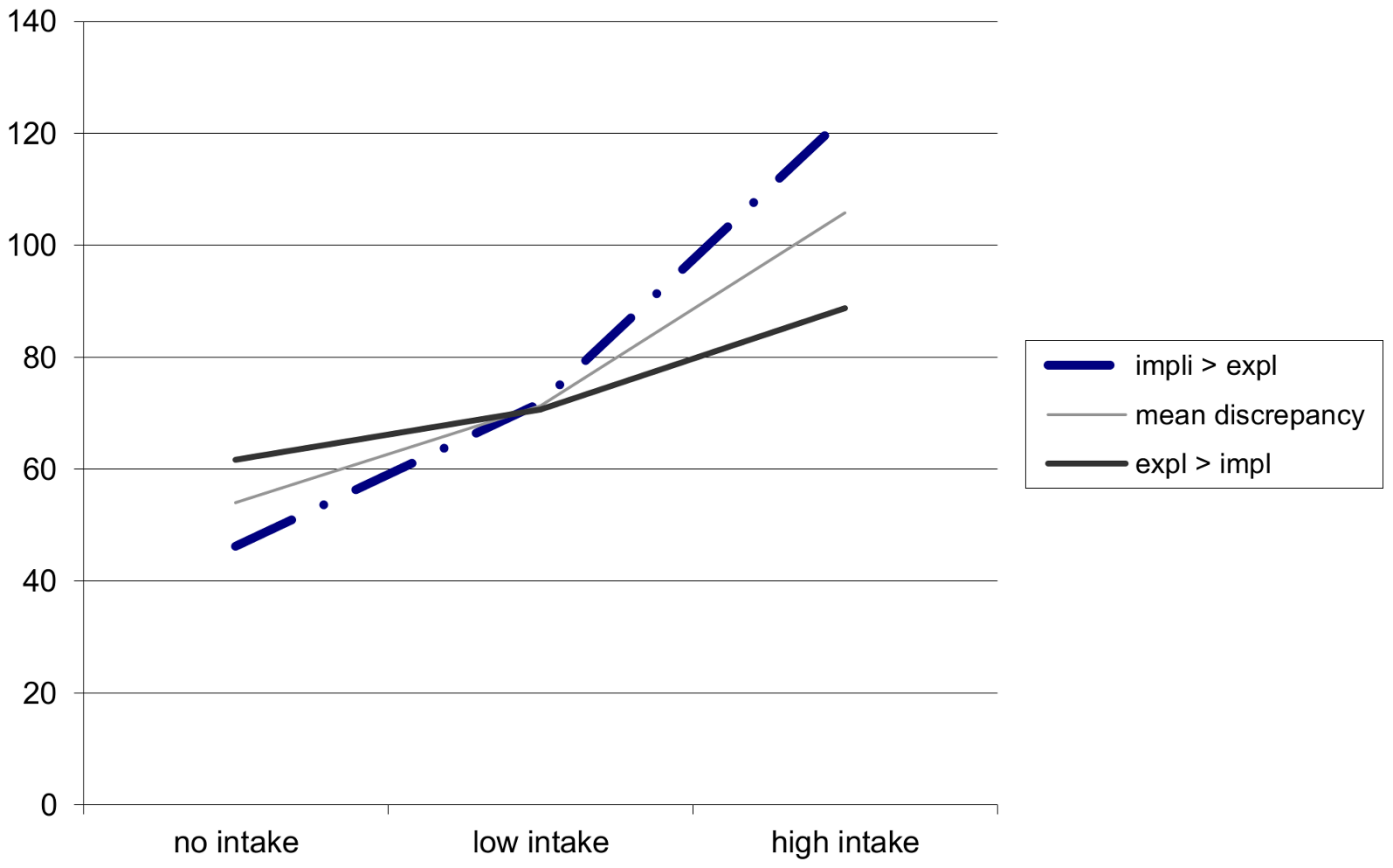
Interaction effect between experimental intake condition and discrepant self-esteem on social modeling of candy intake (kcal). *Note:* The figure presents an interpretation of the interaction effect plotted with the unstandardized regression coefficients. There is a significant difference between the no- and high-, and low- and high-intake condition for youngsters with higher ISE than ESE.

Analyses (N = 113) were performed to further investigate a possible discrepancy between explicit and implicit self-esteem. Consistent with previous research [48], ESE and ISE were not correlated (*r* = .06 *p* = .51). Also, BE and ISE were not correlated (*r* = .08 *p* = .42). To create a single index of discrepant self-esteem, the standardized ISE scores were subtracted from the standardized ESE scores so that higher scores indicate higher ESE and lower ISE. Model 1 revealed a significant difference between the no- versus high-intake condition (*β* = −.24, SE = .08, *p* = .004) but not between the no- versus low-intake condition (*p* = .86). Model 2 revealed that there was a significant difference between the low- and high-intake condition (*β* = −.26, SE = .07, *p*<.0001). Figure 4 illustrates the interpretation of the interaction effect between ESE and ISE. Participants with higher ISE than ESE adjusted more to the remote confederate’s candy intake than participants with higher ESE than ISE.

An additional discrepancy score was computed between BE and ISE (N = 115). Model 1 revealed no significant differences between the no- versus low-intake condition (*p* = .42) or the no- versus high-intake condition (*p* = .11). Model 2 revealed that there was a significant difference between the low- versus high-intake condition (*β* = −.33, SE = .14, *p* = .014). Again, participants with higher ISE than BE adjusted more to the remote confederate’s candy intake than participants with higher BE than ISE.

## Discussion

The present study was the first to investigate whether young teenager’s palatable food intake is affected by peer intake in a social media setting and whether this association was moderated by different types of self-esteem. Findings indicated that youngsters adjusted their food intake to the amount eaten by a peer in an online interaction and that this relation was qualified by body esteem (BE) and implicit self-esteem (ISE). Youngsters with lower BE and higher ISE modeled peer intake. Global explicit self-esteem did not moderate the social modeling effect. In addition, this study was the first to indicate that discrepant self-esteem moderated social modeling behavior. That is, youngsters with so-called “damaged” self-esteem (i.e. higher ISE than ESE) were found to follow peer intake more closely than those with lower ISE than ESE.

Going beyond previous studies on normative influences on food intake by means of remote [9], [10], [11], [12], [13] or real confederates [5], [6], [18], [49], the current findings showed that social modeling behavior can also occur through online interaction. Youngsters modeled their peers when eating nothing compared to something, regardless of the amount of candy (i.e., a modest or substantial). Notably, this modeling pattern is in line with previous findings in normal-weight children who had a confederate physically present in the same room (the study of Bevelander et al. showed that normal-weight children ate similar amounts when a peer ate either a modest or substantial amount of food, whereas overweight children ate similar amounts when a peer ate nothing or a modest amount and increased their intake when a peer ate a substantial amount of food) [18]. It seems that the influence of a peer via social media might be similar to a real-life eating situation. Given that people increasingly engage in social interactions via the Internet, it is relevant to examine the impact of peers on food intake via social media. It should be noted that a previous study in which female students were exposed to an eating remote (video) confederate did not find a modeling effect [50]. The authors suggested that the indication of how much the remote confederate consumed had no effect, because the consumption environment (i.e., task and physical surrounding) differed between the confederate and participants. The current study provided additional insight. Although the tasks were the same, the remote confederate was not in a similar surrounding as the participant. This might indicate that social modeling could be affected by dissimilarity in people’s activities rather than the physical environment. It would be interesting to further investigate this by means of modeling studies in which people perform different tasks versus the same tasks in the same context, for example.

The moderating effects of self-esteem on social modeling behavior were also examined in the present study. In line with the hypothesis, youngsters with lower BE modeled a peer’s candy intake more than those with higher BE; that is, when the peer ate nothing compared to a substantial amount of food. Notably, this moderation effect was not found for ESE. The findings support previous research on the notion that BE as a domain-specific self-esteem might provide more insight into explaining specific behavioral patterns compared to ESE [27]. Thus, body confidence might be more relevant than the general sense of well-being with regard to adjusting to a peer’s food intake. The majority of youngsters appear preoccupied with a slim body image and are often conscious of their weight [51]. It is proposed that youngsters with lower BE are more insecure or experience distress about their body shape in an eating situation with an unknown peer than those with higher BE [52]. As young people often engage in social comparisons, those with lower BE might have followed the intake of a peer to avoid eating inappropriately compared to those with higher BE; especially, when the peer was eating nothing compared to a large amount of food (in youngsters who were satisfied or wanted to lose weight, this was also true for when the peer was eating nothing compared to a modest amount of food).

In contrast to BE and ESE, the findings on the role of ISE on social modeling may seem surprising. Youngsters with higher ISE modeled peer food intake more than those with lower ISE. As this was the first study examining the role of ISE on social modeling behavior, explanations are speculative. Implicit beliefs about the self are proposed to develop at an early age and become fairly stable over time, whereas ESE can fluctuate and, moreover, can differ from ISE [53]. Research on the role of ISE and the connectedness in people’s relationships propose that ISE is associated with the regulation of affiliation responses [54]. Furthermore, ISE is found to manifest in nonverbal behavior (e.g. nodding head affirmatively when someone speaks, smile at someone) and may contribute to the regulation of people’s bonding and affiliation efforts, which might be similar to modeling each other’s behavior [17], [55], [56]. DeHart et al. [54] proposed that implicit self-esteem might function as an indicator of social acceptance. For example, when there is a need to affiliate, ISE is already activated before ESE [57]. In the current study, the youngsters had to engage in a social interaction with an unfamiliar peer, which might have activated their affiliation response. It is speculated that youngsters who possessed higher levels of ISE were more likely to automatically engage in nonverbal behaviors (e.g. modeling) than those with lower ISE. Following this tentative reasoning, ISE might regulate one’s capacity to perform nonverbal social behavior, so those with higher ISE match the food intake of their peers more often than youngsters with lower ISE.

An additional explanation for the findings on explicit and implicit self-esteem might be found in dual process models, which provide a framework for integrating both forms of self-esteem. Previous research found that people suffering from personality or clinical disorders (e.g., narcissism [58], depression and loneliness [59], bulimia nervosa [34]) possessed low ESE while at the same time displaying high ISE. It is suggested that people process information through two separate but possibly interacting systems: a slow conscious reflective mode of processing drawing on cognitive capacity and effortful retrieval of information and a fast automatic mode drawing on associative links in memory. In line with this, ESE is assumed to be a product of the reflective mode, whereas ISE is assumed to be rooted in the associative mode. The incongruity between the explicit reflective and implicit associative self-esteem-systems presents a way to distinguish between two types of self-esteem discrepancies: a combination of high ISE and low ESE (i.e. “damaged” self-esteem or “discrepant low”) versus low ISE combined with high ESE (i.e., “fragile” self-esteem or “discrepant high”) [33], [58]. ISE is suggested to represent the ideal self, whereas ESE represents the real self. A discrepancy between ISE and ESE could consequently lead to a disturbed feeling [35]. Damaged self-esteem may thus be seen as an indicator of psychological distress that can create uncertainty and lead to lower levels of mental health [36]. In this study, youngsters with damaged self-esteem (higher ISE than ESE) were found to follow the food intake of a peer more closely, while those with fragile self-esteem did not. As research on discrepant self-esteem, depression and loneliness suggested that ISE might be indicative of desired social relationships (whereas ESE represents actual social relationships) [59], it is possible that the youngsters engaged in social modeling behavior to fulfill their affiliation goals. As this is the first study to examine the role of implicit and explicit self-esteem on social modeling behavior of eating, more research is warranted to investigate the impact of self-esteem on people’s eating behavior in social contexts. Based on the current findings, it might be relevant to include implicit measures of self-esteem in conceptual models that aim to examine social modeling.

Several limitations associated with the current study are worth mentioning. First, the participant’s affiliation purposes were not measured during their social interaction. Although previous research supports the notion that people want to fulfill their affiliation goals through social modeling, the present study does not provide insight into whether the participants wanted to be liked by their peers. Future studies could code nonverbal behaviors such as eye contact or smiling in order to establish affiliation goals. Second, the homogeneity of the study population can be seen as a limitation. In contrast to implicit self-esteem which stays fairly stable over time, research has shown that age has an effect on explicit self-esteem across the life span [60]. In general, self-esteem is highest during childhood but significantly declines from childhood (ages 9–12) to adolescence (ages 13–17) and continues to decline into the college period (ages 18–22). After this period, self-esteem rises throughout adulthood [60]. It would be interesting to conduct further research on the role of self-esteem in peer modeling among older study populations. In addition, this study consisted out of few overweight or obese youngsters. Future research should concentrate on this weight category as well. Furthermore, this study only involved normal-weight confederates. It would be interesting to investigate whether social modeling would be different within overweight/normal-weight or overweight dyads due to possible different affiliation goals or social norms. Third, the children’s subjective hunger status was measured only after the social interaction to conceal the aim of the study. Another strategy might be to measure the children’s subjective hunger before the study or assess when (or how much) they ate (during) their last meal. Fourth, the remote peer was videotaped, so a real ongoing social interaction was not possible. Qualitative impressions after watching the video recordings of the participants showed that they did not try to verbally contact the peer after a few minutes. Although this seemed to have no effect on modeling behavior, it would be interesting to test social modeling during a real ongoing chat session. Also, the confederates were strangers. As people are more likely to chat with family and friends than with strangers and the influence of strangers on food intake has been shown to be less strong than the influence of familiar peers [61], future studies should investigate the impact of family and friends on food intake via social media interaction. Finally, there is an ongoing debate about the validity of implicit measures to assess implicit self-attitudes. Implicit self-esteem is a complex construct, and different implicit measures may capture distinct aspects of ISE [62]. Therefore, future research is warranted to use multiple indirect measures when implicit self-attitudes are examined, for example, by assessing implicit body esteem.

In conclusion, this study broadens the existing scope of normative influences on young people’s palatable food consumption. To date, we often engage in social contact by social media interactions. As this study found that youngsters even conform to their peer’s food intake via social media, online interactions should also be accounted for in research on the influence of the social environment on food intake or the development of intervention strategies. Given that body image is increasingly important in society, young people with lower body esteem may be more susceptible to peer influences on food intake. In addition, this study provided new insights into the role of self-esteem and people’s adjustment to their peers. Future modeling studies with real confederates should include self-esteem measures.
